# Clinical Features, Etiology, and 6-Month Prognosis of Isolated Corpus Callosum Infarction

**DOI:** 10.1155/2019/9458039

**Published:** 2019-05-14

**Authors:** Zhiyong Zhang, Xiufeng Meng, Wei Liu, Zunjing Liu

**Affiliations:** ^1^Department of Neurology, China-Japan Friendship Hospital, Beijing 100029, China; ^2^Functional Department, Hospital of Tsinghua, Beijing 100084, China

## Abstract

As the largest subcortical commissural fiber, the corpus callosum plays an important role in cerebral functions and has abundant blood supply from bilateral circulation. Isolated corpus callosum infarction (ICCI) may have specific characteristics. The aim of the study is to evaluate the clinical features, etiology, and 6-month prognosis of ICCI. Consecutive patients with acute ICCI treated at the China-Japan Friendship Hospital between June 2012 and June 2016 were retrospectively assessed for clinical and imaging findings. These cases were compared with patients suffering from other isolated supratentorial subcortical infarctions, matched for age, sex, and infarction size (n=60; control group). ICCI etiology and 6-month prognosis were further analyzed. ICCI cases accounted for 2.9% (33/1125) of all acute ischemic strokes and 30 patients were included. Most patients (n=28, 93.3%) presented nonspecific clinical symptoms, and only two (6.7%) with diffuse infarction developed callosal disconnection syndrome (CDS). The splenium was the most frequent site (37.5%). Large artery atherosclerosis (LAA) (n=16, 53.3%) was the most common etiology. Only four (13.3%) patients developed transient ischemic attacks (n=1, 3.3%) or cerebral infarction (n=3, 10%) during the 6-month follow-up. The frequency of good prognosis (modified Rankin score of 1-2 and without cardiovascular events) was higher in patients with ICCI compared with controls (P=0.024). Poor prognosis was associated with multiple cerebrovascular stenosis, diffuse/large infarction, and diabetes (all P<0.05). ICCI is a rare stroke type, frequently involving the splenium; its common etiology is likely LAA. Most patients show nonspecific symptoms, with only a few developing CDS. ICCI generally shows favorable short-term outcome.

## 1. Introduction

The corpus callosum (CC) connects the left and right nerve fiber bundles [1, 2]. The CC is divided into the rostrum, genu, body, and splenium and is supplied by multiple vessels involving both the anterior and the posterior circulations [2]. Due to its abundant blood supply, ischemic stroke at this site is relatively rare [3]. Isolated CC infarction (ICCI) is considerably rare, with clinical manifestations and prognosis commonly overshadowed by concurrent infarctions at other sites [4]. The exact epidemiology of ICCI is unknown, but Li et al. [5] reported 59 patients (3.6%) with CC involvement, including seven cases of ICCI (0.4%), among 1629 patients with cerebral infarction.

Hemisphere dysfunction and interhemispheric disconnection symptoms may occur upon lesions of the CC [6–9]. The most classic symptom is known as the callosal disconnection syndrome (CDS), which encompasses apraxia, agraphia, left-hand tactile anomia, and the alien hand syndrome (AHS) [10]. The two largest series of CC infarction (not necessarily isolated) so far reported cognitive abnormality (40%), language disorder (48%), forced laughter and crying (20%), and limb dyskinesia (84%) [5], as well as hemiparesis (73%), sensory abnormality (32%), ataxia (10%), aphasia (17%), dysarthria (22%), AHS (3%), and disturbance of consciousness (10%) [4]. CDS caused by CC infarction has been reported [11–13]. It remains unclear how infarct of the CC, a very important subcortical structure, differs from other subcortical infarcts.

A better knowledge of the clinical features of ICCI should help improve the diagnostic and prognostic accuracy. Unfortunately, studies addressing this issue are scarce. Therefore, the present study aimed to retrospectively analyze and discuss the clinical characteristics, etiology, and prognosis of the ICCI cases overobserved at a single center over 4 years, exploring this special and rare type of stroke in detail.

## 2. Materials and Methods

### 2.1. Study Design and Patients

This was a retrospective study of patients with acute ICCI identified from a registry of consecutive patients with acute ischemic stroke and regular follow-up at the Department of Neurology of the China-Japan Friendship Hospital between June 2012 and June 2016. Acute ischemic stroke was diagnosed according to World Health Organization criteria [14], with magnetic resonance imaging (MRI) confirmation. The study was approved by the ethics committee of the China-Japan Friendship Hospital. The need for individual consent was waived by the committee because of the retrospective nature of the study.

Patients were included in the ICCI group if they presented new infarction limited to the CC, without involvement of other sites. The exclusion criteria were (1) no available cerebrovascular morphologic imaging data during hospitalization, either by magnetic resonance angiography (MRA), computerized tomography angiography (CTA), or digital subtraction angiography (DSA) or (2) loss to follow-up or incomplete follow-up data.

The control group included patients from the same registry and with new infarctions occurring in supratentorial subcortical areas except the CC. The diagnostic criteria for subcortical infarction referred to the classification by Donnan et al. [15]. Ultimately, control patients were matched 2:1 based on age (within 2 years), sex, and infarction maximum diameter (within 15%) to the ICCI group.

Upon admission and routine practice, all patients underwent routine examination, comprehensive screening of the risk factors for cerebrovascular diseases, cerebrovascular examinations, and stroke etiology assessment based on the Trial of ORG 10172 in Acute Stroke Treatment (TOAST) criteria including large artery atherosclerosis (LAA), cardioembolism, small artery occlusion, other determined causes, and undetermined etiology [16]. For patients with undetermined stroke etiology or for patients with a possibility of having other diseases, cranial high-resolution MRI (HR-MRI), routine electrocardiography, 24-h Holter monitoring, echocardiography, transcranial Doppler, transesophageal ultrasound, electroencephalography, electromyography, specific clinical tests, and cerebrospinal fluid examination were performed to exclude other diseases and refine the differential diagnosis.

### 2.2. Data Collection

Clinical data during hospitalization and follow-up were collected from the stroke registry database. Hospitalization items included general information (age and gender), risk factors for cerebrovascular diseases (hypertension, diabetes, hyperlipidemia, hyperhomocysteinemia, history of stroke, coronary heart diseases, atrial fibrillation, smoking, alcoholism, and a family history of atherosclerotic cardiovascular diseases), clinical manifestations, National Institutes of Health Stroke Scale (NIHSS) score at admission and discharge, and stroke etiology.

### 2.3. Imaging

Imaging data were collected using the picture archiving and communication system (PACS) (Neurosoft 3.0, Shenyang, China) in our hospital. The infarction maximum diameter was measured using the MRI built-in software. The images were reviewed and analyzed by two experienced neuroradiologists. The imaging findings were based on the MRI examination and evaluated as (1) location of the primary callosal infarction (genu, body, splenium, or mixed location; since the rostrum is relatively small and difficult to assess by MRI, it was not included); (2) size of the infarction and divided into large (maximum diameter ≥15 mm) or small (maximum diameter <15 mm); and (3) presence of stenosis or occlusion in cranial large vessels and identification of the culprit vessel.

### 2.4. Follow-Up

After discharge, all patients underwent routine follow-up for at least 6 months at the stroke outpatient clinic, at one-month intervals. Follow-up included Modified Rankin score (mRS) at 6 months after discharge and cardiovascular events (transient ischemic attack (TIA), new cerebral infarction, cerebral hemorrhage, subarachnoid hemorrhage, myocardial infarction, and all-cause death).

### 2.5. Statistical Analysis

Statistical analysis was performed using SPSS 17.0 (IBM, Armonk, NY, USA). Continuous variables are expressed as means ± standard deviation and were analyzed using Student's* t*-test or the paired* t*-test for repeated measures. Categorical variables are presented as frequencies and analyzed using the chi-square test or the McNemar test for repeated measures. Two-sided P values <0.05 were considered statistically significant. The Bonferroni correction was applied for multiple comparisons, with a P value of <0.05/n being considered statistically significant.

## 3. Results

### 3.1. Patient Characteristics

**Figure 1:**
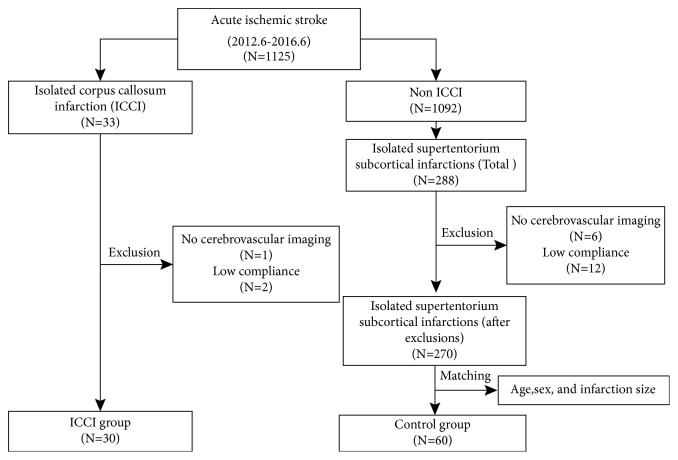
Study flowchart.

Thirty-three (2.9%) patients with acute ICCI were identified from the 1125 patients with acute stroke registered at the China-Japan Friendship Hospital between June 2012 and June 2016. Based on the exclusion criteria, one patient was excluded because of no available cerebrovascular morphologic imaging data during hospitalization, and two were excluded because of loss to follow-up or incomplete follow-up data. Therefore, 30 consecutive patients were included in the ICCI group. Among the 1125 patients, 288 were identified as being with isolated supratentorial subcortical infarctions. Of these 288 cases, 18 were excluded (6 for lack of imaging data and 12 for loss to follow-up). Among the remaining 270 patients, 60 were matched for age, sex, and infarction size to the ICCI group (see Figure 1).

Table 1 presents the characteristics of the two groups. In the ICCI group, the sites of callosal infarction: gene in 5 cases (16.7%), body in 4 (13.3%), splenium in 14 (46.7%), and mixed location in 7 (23.3%). The numbers of left, right, and bilateral lesions were 8, 18, and 4, respectively. Most patients (28, 93.3%) with ICCI showed nonspecific clinical symptoms. Only two (6.7%) patients with combined splenium, body, and genu infarction developed typical CDS.

**Figure 2:**
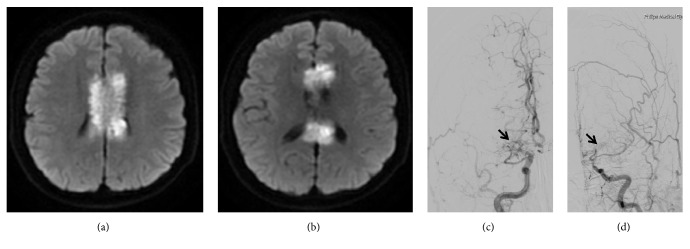
*Male, 38 years, diffusive callosal infarction with the alien hand syndrome caused by Moyamoya disease.* Diffusion-weighted imaging shows an extensive infarction involving the genu, body, and splenium of bilateral corpus callosum (a-b). Digital subtraction angiography demonstrates severe stenosis or occlusion of the terminal segments of bilateral internal carotid arteries, as well as proximal segments of bilateral anterior cerebral arteries and middle cerebral arteries with formation of Moyamoya vessels (black arrows) (c-d).

**Figure 3:**
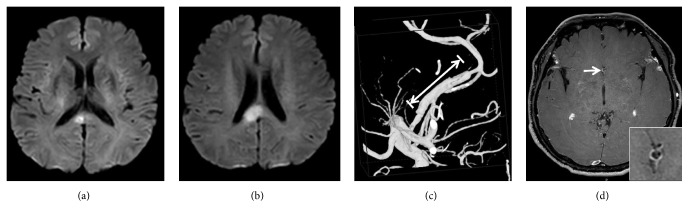
*Male, 53 years, callosal infarction caused by anterior cerebral artery dissecting aneurysm.* Diffusion-weighted imaging (DWI) shows acute infarction in the right corpus callosum splenium (a-b). Three-dimensional digital subtraction angiography (DSA) confirms long segmental dilatation of the right anterior cerebral artery (ACA) (long white arrow) with distal concentric stenosis (c). Axial contrast-enhanced high-resolution magnetic resonance imaging (MRI) clearly reveals the enhanced wall of the ACA and an intima flap in the arterial lumen forming the "double-lumen sign" (white arrow) (d).

**Figure 4:**
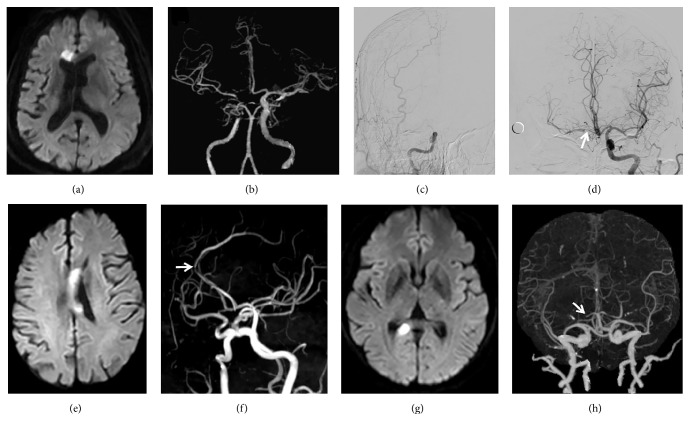
*Infarctions at different sites of the corpus callosum and their affected vessels.* (a-d) Male, 69 years, left hemiparesis. Diffusion-weighted imaging (DWI) shows a new infarction of the right genu (a). Computed tomographic angiography (CTA) and digital subtraction angiography (DSA) reveal complete occlusion of the cavernous segment of the right internal carotid artery, collateral compensation from the left to the right anterior circulation via the anterior communicating artery, and severe stenosis of the A1 segment of right anterior cerebral artery (white arrow) (b-d). (e-f) Male, 75 years, right hemiplegia. DWI shows a new infarction of the left body (e). Magnetic resonance angiography (MRA) shows that the affected vessel is the severe stenotic pericallosal artery (white arrow) (f). (g-h) Female, 57 years, dizziness. DWI shows an isolated infarction of the right splenium (g). CTA shows that the P1 segment of right posterior cerebral artery has occluded (white arrow) (h).

According to the TOAST criteria [14], there were 16 cases (53.3%) caused by LAA, five (16.7%) by cardioembolism, four (13.3%) by small artery occlusion, four (13.3%) by other determined causes, and one (3.3%) by undetermined etiology. Compared with the control group, no significant difference was observed (all P>0.05) (see Table 1). Other rare causes included Moyamoya disease (n=2, 6.7%) (see Figure 2), intracranial artery dissecting aneurysm (n=1, 3.3%) (see Figure 3), and cryptococcal meningitis (n=1, 3.3%). LAA remained a relatively common cause of infarction in different locations, respectively, accounting for 60.0% (3/5), 50.0% (2/4), 42.9% (6/14), and 71.4% (5/7) of the cases of genu, body, splenium, and mixed infarction. The culprit vessels for infarcts at different sites are shown in Table 2 and Figure 4. These findings suggested that cerebrovascular atherosclerotic stenosis was common in the anterior cerebral artery (ACA)-A1/A2 and posterior cerebral artery (PCA)-P1/P2 segments in ICCI.

### 3.2. Characteristics of Three Cases with Diffuse Callosal Infarction

Only three (10.0%) patients with ICCI showed diffusive infarction extending from the genu to the splenium. Their clinical and imaging data are shown in Table 3. The clinical features included relatively serious clinical symptoms, multiple stenosis in both anterior and posterior circulations, and a high recurrence rate (2/3, 67%). As for etiology, two (6.7%) and one (3.3%) patients had LAA and Moyamoya disease cases, respectively (see Figure 2).

### 3.3. Possible Factors Associated with Prognosis

Most patients (25, 83.3%) with ICCI had mRS of 0-2, and only five (16.7%) showed mRS >2 (all with mixed infarction) at 6 months after discharge. In addition, four (13.3%) patients with ICCI experienced cardiovascular events during the 6-month follow-up: one (3.3%) patient developed TIA and three (10.0%) suffered from new cerebral infarction. Overall, based on the mRS and the occurrence of cardiovascular events, most patients (22, 73.3%) had a favorable outcome, while eight (26.7%) showed an unfavorable outcome of ICCI. The short-term prognosis in the ICCI group was better than that of the control group (P=0.024) (Table 1). Multiple cerebrovascular stenosis (P=0.012), diffuse/large infarction (P=0.007), and diabetes (P=0.035) were associated with poor prognosis (Table 4).

## 4. Discussion

Anatomically, the CC is composed of the rostrum, genu, body, and splenium. As the largest subcortical commissural fiber, the CC plays an important role in cerebral functions and has abundant blood supply from bilateral circulation. Therefore, ICCI may have specific characteristics. Nevertheless, the exact features of ICCI remain poorly known. Therefore, this study aimed to evaluate the clinical features, etiology, and 6-month prognosis of ICCI.

In this study, ICCI accounted for only 2.9% of all acute ischemic stroke cases registered at the China-Japan Friendship Hospital between June 2012 and June 2016, confirming its low incidence [4, 5]. Previous studies suggested that callosal infarction commonly occurred in the splenium [5, 17], but discrepant findings have been reported [18]. In this study, splenium infarction frequency was the highest, which might be related to the high infarction incidence in the PCA feeding area compared with that in the ACA [17], racial differences [5], and sample size.

The CC receives rich blood supplies from the anterior and posterior circulations. Despite the presence of vascular variations, the main feeding arteries of CC are the subcallosal artery, median callosal artery, pericallosal artery (PA), and posterior pericallosal artery (PPA) [3, 19]. The subcallosal artery and median callosal artery originate from the anterior communicating artery and occasionally from the ACA as an anatomical variation in some patients, which supply the rostrum, genu, and a small part of body [3, 19, 20]. The PA is a continuation of the ACA, with a long course [3, 19–21]. It is the dominant arterial supply for the body of the CC; the PA has four branches that supply the CC including the cingulocallosal artery, long callosal artery, recurrent cingulocallosal artery, and callosal artery [3]. The callosal artery is also called short callosal artery by Ugur et al. [19]. The PPA is the distal branch of the PCA, mainly supplying the splenial portion of the CC. There are anastomoses between the PA and PPA near the tip of the splenium [3, 19, 20]. Due to the special anatomical structure and different feeding arteries, ICCI etiology might differ among various sites, with atherosclerosis being a common etiology for genu and body, while embolism is more frequent for splenium [18, 22, 23]. In this study, there was no etiological difference between ICCI and other subcortical infarcts, possibly due to the limited sample size. Nevertheless, LAA was a relatively common etiology of ICCI, regardless of the area. This may be because the present study only included isolated lesion at the CC, while previous reports mainly involved patients with multiple infarcts [4, 5]. In addition, the patients in the present study were older and had more risk factors than in previous studies [4, 5]. Anatomically, the ACA is divided into five segments (A1-A5). Anterior communicating artery is from the junction of A1 and A2 segment, and the PA starts from the A4 segment. The PCA is divided into four segments (P1-P4), and the PPA frequently arises from the P3 segment or farther vessel branch [3, 19]. Although the supply vessels of CC are as described above, in case of LAA stroke, the culprit artery may be the parent artery or the proximal artery of the supply artery [24]. In the present study, we found that for CC infarction due to LAA, stenosis of the ACA-A1/A2 and PCA-P1/P2 segments was the most frequent. This stroke mechanism may be caused by hypoperfusion, decreased embolus clearance, or arterial-arterial embolization embolism of the downstream feeding artery of CC due to stenosis of the parent artery. In this study, 13.3% of strokes in the ICCI group were due to small artery occlusion, compared with 31.7% in the control group. Although there was no significant difference (P=0.060) between the two groups, the ICCI group had a lesser tendency to develop small artery occlusion. It may be because the CC may be able to tolerate some degree of small vessels ischemia due to adequate blood supply [17, 25, 26].

Other uncommon etiologies previously reported included carotid artery dissection [27], arteritis [23, 28], Moyamoya disease [29, 30], and variation or dysplasia of the circle of Willis [22, 26]. Bilateral or diffuse callosal infarction most likely involves other intracranial parts; therefore, isolated lesions rarely occur [5, 23]. Common cerebrovascular lesions are severe stenosis or occlusion of bilateral carotid arteries [31–33], but severe stenosis of unilateral PA has also been also reported [34]. Previous studies reported that cryptococcal meningitis could lead to ischemic stroke of deep small vessels, often involving the basal ganglia, internal capsule, and thalamus [35, 36]. A few infarctions involving multiple large vessels have been observed [36], but involvement of the splenium only has not been reported. In this study, Moyamoya disease and ACA dissection were detected, in addition to cryptococcal meningitis. Although ischemic cerebral stroke is usually the onset pattern of Moyamoya disease in adults, ICCI caused by Moyamoya disease is rare and only a few cases have been reported [29, 30]. The etiology of rare infarctions such as ICCI could be mainly abnormal hemodynamics caused by atherosclerosis [18, 31, 32], but it could be also due to cardioembolism [23, 37], tuberculous arteritis, or Takayasu arteritis [18]. In this study, severe stenosis or occlusion of multiple intracranial vessels in bilateral circulations was observed in three patients. According to our analysis, infarct extension from front to rear, crossing the junctional area and involving the genu, body, and splenium, often reflects insufficient collateral compensation between the anterior and posterior circulations. In the present study, the etiology of severe callosal infarction not only included severe arteriosclerosis, but also Moyamoya disease. Besides, since diffuse infarction is often combined with severe intracranial vascular lesions, such cases are highly susceptible to stroke recurrence, as suggested above. Overall, these findings revealed no overt etiological differences between the ICCI and control groups.

As for symptoms, previous studies have suggested that since most patients have limited lesions or a combination of infarcts in other areas, the clinical manifestations are often nonspecific or overshadowed [4, 5, 18]. Likewise, in the present study, most patients had limited lesions and displayed nonspecific clinical features. Since callosal infarction would impact the integrity of neurofibers connecting the two cerebral hemispheres, patients may display loss of interhemispheric contact, which is also referred to as the CDS [13]. The AHS, a psychomotor disorder characteristic of the CDS, manifests as unconscious movements of the affected hand or incoordination or competitive conflict between both hands [38, 39]. In the present study, only two patients showed the AHS; they had diffuse lesions involving the genu, body, and splenium, with the largest infarcts among all 30 subjects, suggesting that the presence of AHS could be a sign of severe ICCI. These findings and previous reports collectively suggest that patients might develop the CDS only with diffuse or bilateral callosal infarct, since this might significantly impact the connection between both hemispheres [5].

In this study, we found that patients with ICCI had better 6-month prognosis than patients with other isolated supratentorial subcortical infarctions. Indeed, the ICCI group showed a higher frequency of mRS score of 1-2 and a lower frequency of cardiovascular s events. Compared to most supratentorial structures, the CC has abundant blood supply and multiple collateral circulations; therefore, rapid compensation is achieved and the neurologic deficits were not serious after ischemia [5]. Besides, because it is composed of arcuate fibers, the CC would mainly undergo demyelination rather than neuronal degeneration or necrosis after infarction [1, 7]. Thus, ischemia would be improved after treatment and rapid remyelination would promote short-term recovery. Further analysis also demonstrated that poor prognosis might be associated with multiple cerebrovascular stenosis, diffusive or large infarction, and diabetes.

To the best of our knowledge, this study has the largest number of patients with ICCI in the literature. Nevertheless, the sample size was still small. Other limitations of this study include the single-center retrospective methodology, inclusion and exclusion criteria, and a relatively short follow-up period, which may have introduced bias. Therefore, future multicenter prospective studies are required for a better understanding of ICCI. Additional studies with larger groups of patients are necessary to confirm these results and would allow multivariable analyses.

## 5. Conclusions

In conclusion, ICCI is a rare cerebrovascular event, frequently involving the splenium. The common etiology of ICCI is LAA, and common vascular lesions occur at ACA-A1/A2 and PCA-P1/P2 segments. Most patients show nonspecific symptoms, with only few developing CDS, which could indicate diffuse lesions involving the whole CC. In addition, this study reveals a good 6-month prognosis for ICCI and some factors affecting prognosis, including multiple cerebrovascular stenosis, diffusive or large infraction, and diabetes. These characteristics not only help understand the special types of subcortical stroke, but also provide some valuable references for future clinical studies.

## Figures and Tables

**Table 1:** Characteristics of the patients in the ICCI and control groups.

Variable	ICCI	Control	P
	(n=30)	(n=60)	
Age, median (range), years	64 (38-80)	63 (38-79)	-* *-* *-
Male sex, %	63.3	63.3	-* *-* *-
Infarction maximum diameter, median (range), mm	14.5 (8-90)	15.2 (9-88)	-* *-* *-
Atherosclerotic risk factors, n (%)			
Hypertension	22 (73.3%)	40 (66.7%)	0.520
Diabetes	15 (50.0%)	26 (43.3%)	0.549
Hyperlipidemia	10 (33.3%)	24(40.0%)	0.539
Hyperhomocysteinemia	6 (20.0%)	18 (30.0%)	0.312
History of stroke	9 (30.0%)	15 (25.0%)	0.613
Coronary artery disease	5 (16.7%)	9 (15.0%)	0.837
Atrial fibrillation	3 (10.0%)	14 (23.3%)	0.128
Smoking	7 (23.3%)	20 (33.3%)	0.329
Alcoholism	6 (20.0%)	15 (25.0%)	0.597
Family history of vascular diseases	6 (20.0%)	17 (28.3%)	0.393
Stroke etiology*∗*			
Large artery atherosclerosis	16 (53.3%)	20 (33.3%)	0.068
Cardiac embolism	5 (16.7%)	12 (20.0%)	0.703
Small artery occlusion	4 (13.3%)	19 (31.7%)	0.060
Other determined causes	4 (13.3%)	6 (10.0%)	0.906
Undetermined	1 (3.3%)	3 (5.0%)	1.000
Short-term prognosis			
Favorable outcome	22 (73.3%)	29 (48.3%)	0.024

*∗*P<0.01 (0.05/5) was considered statistically significant by Bonferroni correction.

**Table 2:** The culprit vessels of different infarct sites for 16 patients with LAA etiology.

Site (n)	Affected vessels (n)				
Genu (3)	ACA-A1 (2)	ACA-A2 (1)			
Body (2)	ACA-A1 (1)	PA (1)			
Splenium (6)	PCA-P1 (3)	PCA-P2 (2)	ACA-A2 (1)		
Mixed (5)	B-ACA-A1+ PCA-P1 (1)	B-ACA-A1+ BA (1)	B-ACA-A1 (1)	PCA-P2 (1)	ICA-C7 (1)

ACA: anterior cerebral artery, PA: pericallosal artery, PCA: posterior cerebral artery, PPA: posterior pericallosal artery, BA: basilar artery, ICA: internal carotid artery, B: bilateral.

**Table 3:** Clinical and imaging features of three patients with diffuse callosal infarction.

Age/sex/side	Manifestations	Cerebrovascular lesions	Etiology	Follow-up
M/62/B	Hemiplegia, aphasia	O: R-ACA-A1;	LAA	mRS = 3
		S:L-ACA-A1, BA;		
		M: B-MCA-M1		
M/38/B	AHS	O: R-MCA-M1;	MD	RS (R-AC)
		S:B-ICA-C7, B-ACA-A1;		
		M:L-MCA-M1;		
		Moyamoya vessels		
F/57/R	AHS, aphasia	O: L-ACA-A1, L-ICA-C1;	LAA	RS (R-AC)
		S:R-ACA-A1,L-PCA-P1,L-VA-V1;		
		M: L-MCA-M1		

B: bilateral, R: right, L: left, AHS: alien hand syndrome, O: occlusion, S: severe stenosis, M: moderate stenosis, ACA: anterior cerebral artery, BA: basilar artery, MCA: middle cerebral artery, ICA: internal carotid artery, PCA: posterior cerebral artery, VA: vertebral artery, LAA: large artery atherosclerosis, MD: Moyamoya disease, mRS: modified Rankin Scale, RS: stroke recurrence, AC: anterior circulation.

**Table 4:** Comparison of the possible related factors between good and poor prognosis.

	Good prognosis (mRS=1-2)	Poor prognosis (mRS >2 or adverse event)	P
Multiple cerebrovascular stenosis	31.8% (7)	31.8% (7)	0.012
Diffuse/large infraction	18.2% (4)	75.0% (6)	0.007
NIHSS ≥6 at admission	13.6% (3)	50.0% (4)	0.060
Hypertension	68.2% (15)	87.5% (7)	0.391
Diabetes	36.4% (8)	87.5% (7)	0.035
Hyperlipidemia	27.3% (6)	50.0% (4)	0.384
Hyperhomocysteinemia	13.6% (3)	37.5% (3)	0.300
History of stroke	22.7% (5)	50.0% (4)	0.195
Coronary artery disease	13.6% (3)	25.0% (2)	0.589
Atrial fibrillation	9.1% (2)	12.5% (1)	1.000
Smoking	18.2% (4)	37.5% (3)	0.345
Alcoholism	18.2% (4)	25.0% (2)	0.645
Family history of vascular diseases	18.2% (4)	25.0% (2)	0.645
≥3 risk factors	54.5% (12)	87.5% (7)	0.199

Multiple cerebrovascular stenosis: ≥3 large cranial vessels with at least moderate stenosis.

## Data Availability

The data used to support the findings of this study are available from the corresponding author upon request.
